# JSBML 1.0: providing a smorgasbord of options to encode systems biology models

**DOI:** 10.1093/bioinformatics/btv341

**Published:** 2015-06-16

**Authors:** Nicolas Rodriguez, Alex Thomas, Leandro Watanabe, Ibrahim Y. Vazirabad, Victor Kofia, Harold F. Gómez, Florian Mittag, Jakob Matthes, Jan Rudolph, Finja Wrzodek, Eugen Netz, Alexander Diamantikos, Johannes Eichner, Roland Keller, Clemens Wrzodek, Sebastian Fröhlich, Nathan E. Lewis, Chris J. Myers, Nicolas Le Novère, Bernhard Ø. Palsson, Michael Hucka, Andreas Dräger

**Affiliations:** ^1^European Bioinformatics Institute (EBI), Hinxton, UK,; ^2^Babraham Institute, Babraham Research Campus, Cambridge, UK,; ^3^University of California, San Diego, La Jolla, CA, USA,; ^4^Novo Nordisk Foundation Center for Biosustainability, University of California, San Diego, La Jolla, CA, USA,; ^5^The University of Utah, Salt Lake City, UT, USA,; ^6^Marquette University, Milwaukee, WI, USA,; ^7^The University of Toronto, Toronto, ON, Canada,; ^8^Boston University, Boston, MA, USA,; ^9^Center for Bioinformatics Tuebingen (ZBIT), University of Tuebingen, Tübingen, Germany,; ^10^Tel Aviv University, Tel Aviv, Israel,; ^11^Roche Pharmaceutical Research and Early Development, pRED Informatics, Roche Innovation Center, Penzberg, Germany,; ^12^Leibniz Institute of Plant Genetics and Crop Plant Research (IPK), Gatersleben, Germany,; ^13^Martin Luther University of Halle-Wittenberg, Halle (Saale), Germany and; ^14^The California Institute of Technology, Pasadena, CA, USA

## Abstract

**Summary:** JSBML, the official pure Java programming library for the Systems Biology Markup Language (SBML) format, has evolved with the advent of different modeling formalisms in systems biology and their ability to be exchanged and represented via extensions of SBML. JSBML has matured into a major, active open-source project with contributions from a growing, international team of developers who not only maintain compatibility with SBML, but also drive steady improvements to the Java interface and promote ease-of-use with end users.

**Availability and implementation**: Source code, binaries and documentation for JSBML can be freely obtained under the terms of the LGPL 2.1 from the website http://sbml.org/Software/JSBML. More information about JSBML can be found in the user guide at http://sbml.org/Software/JSBML/docs/.

**Contact:** jsbml-development@googlegroups.com or andraeger@eng.ucsd.edu

**Supplementary information:** Supplementary data are available at *Bioinformatics* online.

## 1 Introduction

The Systems Biology Markup Language (SBML) is a widely used format that enables easy distribution of systems biology data, models and diagrams, and it allows the easy exchange of data and models between a variety of software systems (Hucka *et al.*, 2003; Dräger and Palsson, 2014). Given the wide coverage of the latest SBML version, it is unsurprising that the standard is relatively complex. An appropriate computational architecture greatly simplifies the work that software developers need to do in order to support importing and storing SBML-based information for computational analysis. JSBML (Dräger *et al.*, 2011) is the official, pure Java-based *application programming interface* (API) library for SBML. It enables systems biology information to be expressed in Java data structures patterned after the SBML format for fast access. JSBML 1.0 implements the ability to encode, exchange and use all parts of SBML, up to the current release, SBML Level 3 (L3), including all community-approved (and several prototype) SBML packages for L3, as described later.

One important aspect of SBML is its ability to provide additional capabilities to encode specific types of systems biology models. These capabilities are extensions of the core SBML L3 format and are known as ‘packages’. Although many models can be fully represented using only the core set of SBML constructs, these extensions support (i) other model features that cannot be formulated with the SBML core standards and (ii) additional constructs that enable users in specific fields to formulate, interface and use the SBML framework more easily within their modeling approaches. SBML packages enable the ability to build models that encompass several formalisms within one overall framework. Several tools today use JSBML 1.0 in this way and encode multiple types of systems biology models; examples include iBioSim (Madsen *et al.*, 2012), KEGGtranslator (Wrzodek *et al.*, 2011) and GINSim (Gonzalez Gonzales *et al.*, 2006). In addition, because JSBML is built to be an interpreter for SBML, this functionality, represented by a single data structure, can easily be embedded into existing Java programs.

Since its inception, JSBML has fostered a community of active developers who aim to provide regular code updates, provide major and minor bug fixes to releases, and partake in discussions on standards for the systems biology modeling community (COMBINE, Waltemath *et al.*, 2011a). These community interactions have helped improve JSBML substantially since its launch.

## 2 Results

### 2.1 Improvements

JSBML’s first public release was in 2011 (Dräger *et al.*, 2011) and it has since undergone considerable change and expansion. A major goal of the initial release was to present a software package which differentiated itself from libSBML, JSBML’s C++ language counterpart (Bornstein *et al.*, 2008), and its Java-language bindings. Since then, JSBML has maintained compatibility with SBML and libSBML and has introduced extra functionalities.

In addition to support for SBML L3 core and packages, JSBML 1.0 has incorporated improvements to software efficiency. For instance, improvements to internal interfaces now speed up model input/output operations. The different identifier namespaces in SBML are now managed with an IdManager interface which is able to reconcile redundant identifiers among packages. A new Math infix parser is able to mimic the same behavior as the libSBML L3 Math parser and can handle complex mathematical formulas. The manipulation and merging of Units in SBML has also been greatly improved.

Also, several features have been added to JSBML 1.0 to improve end-user convenience and accessibility. For instance, various utility methods have been added that allow users to manipulate JSBML’s in-memory data object. Furthermore, user-defined objects can be temporarily added to the JSBML data structure for any model component. XML annotations in SBML are read as XMLNode object instead of Strings, making it easier to manipulate non-standard annotations in SBML. Advanced logging functionalities via the Apache log4j project allows users to monitor JSBML actions.

Finally, JSBML has been better integrated with other software such as Apache Maven, a dependency management tool, BioJava 3 (Prlić *et al.*, 2012), a bioinformatics toolbox, and CellDesigner (Funahashi *et al.*, 2008), a biochemical network modeling and visualization tool.

### 2.2 Support for SBML packages

#### 2.2.1 Approved packages

At the time of writing, all approved SBML L3 extensions are supported by JSBML 1.0. We describe the support below, and provide insights into the mapping between modeling formalisms and the corresponding JSBML data objects. An abbreviated version of the JSBML class hierarchies are presented in Supplementary Figure S1. The JSBML User Guide has figures that lay out the JSBML class hierarchy for each SBML package, displaying the full capabilities of the JSBML data objects that encode each package. In Table 1, approved SBML packages constitute the first four rows. The *Qualitative Models* package (**qual**, for short) allows species in a model to have non-quantitative or non-continuous levels (Chaouiya *et al.*, 2013). This may manifest as Boolean or discrete values, and is primarily employed in modeling gene regulation, signaling pathways, logical/Boolean networks (Schmulevich *et al*., 2002), and Petri nets (Breitling *et al.*, 2008). *Flux Balance Constraints* (**fbc**, Olivier and Bergmann, 2013) encodes components for constraints-based modeling (Lewis *et al.*, 2012), which employs a class of models in which the canonical stoichiometric relations between reactions and metabolites are specified as constraints for mathematical optimization. *Layout* provides the ability to encode graphical information for model diagrams. The structure for this extension mirrors the SBML core hierarchy by introducing graphical counterparts to reactions and species. The fourth approved package, *Hierarchical Model Composition* (**comp**) provides a generic framework to encode models as hierarchical entities in SBML (Smith *et al.*, 2013). JSBML’s **comp** implementation provides access to elements within sub-models and interfaces with other models.

**Table 1.** SBML Package status

Name	Id	Description	JSBML support
Qualitative models	qual	Qualitative values for species	Full
Flux balance constraints	fbc	Constraints based parameters	Full
Layout	layout	Network layout topology	Full
Hierarchical model composition	comp	Modular, hierarchical entities	Full
Spatial processes	spatial	Location and geometries	Full
Groups	groups	Grouping elements	Full
Arrays	arrays	Values and entities in arrays	Full
Required elements	req	Required model elements	Full
Distributions	distrib	Model values as statistical distributions	Full
Dynamic structures	dyn	Dynamic model entities	Full
Rendering	render	Network layout style	Full
Multistate and multicomponent species	multi	Rule based modeling	Partial

#### 2.2.2 Draft packages

Draft specifications are available for the remaining SBML packages; they are encoded in JSBML with varying maturity. JSBML fully supports the current specifications of seven packages whose community approvals are pending: *Spatial Processes* (**spatial**, Schaff *et al.*, 2014) specifies geometric descriptions of biochemical models’ components using a cellular coordinate system that can describe non-uniform molecular distributions, diffusive transport and spatially localized reactions; *Groups* (**groups**, Hucka and Smith, 2013) agglomerates SBML model elements and can be linked to annotations and SBO terms (Courtot *et al.*, 2011) to contextualize sets of objects for other programmers and modelers; *Arrays* (**arrays,** Watanabe *et al.*, 2013) extends SBML variables to include arrays of values, thereby representing repeated or regular model structures more efficiently; *Required Elements* (**req**, Smith and Hucka, 2013) allows a model to indicate which components have had their mathematical meanings changed by (e.g.) the use of another SBML package; *Distributions* (**distrib**, Moodie and Smith, 2013) encodes statistical distributions and their sampling; *Dynamic Structures* (**dyn**, Gomez *et al.*, 2014), which supports the definition of dynamical behaviors for model entities; and *Rendering* (**render**, Gauges *et al.*, 2011), used in conjunction with **layout** to provide symbol and style information for diagrams. The last package, *Multistate and Multicomponent Species* (Zhang and Meier-Schellersheim, 2013) is still under development. The JSBML project is committed to support all SBML packages as their specifications come out and are deemed stable by the community.

#### 2.2.3 JSBML and package formation

Some package specifications have been influenced by JSBML development. For example, protocols for validation and flattening of array constructs in the **arrays** package. Prior to the development of **arrays** in JSBML, the specification lacked important validation rules that serve as quality controls for math operations done with **arrays** data structures. JSBML also provided the environment to ensure **arrays** was compatible with other SBML L3 packages, helping to build a more robust specification. Finally, JSBML development of the **arrays** package was used to generate simulatable examples for the SBML L3 **arrays** specification. Therefore, JSBML provides a means to test and implement new package development for future versions of SBML.

## 3 Conclusions

JSBML version 1.0 marks the maturation of this software library as an essential component for any systems biology pipeline that runs in the Java Virtual Machine, and joins other biological exchange format interpreters (Paxtools, Demir *et al.*, 2013; CellML API, Miller *et al.*, 2010; SED-ML, Waltemath *et al.*, 2011b; SBOL, Galdzicki *et al.*, 2014; libSBGN, van Iersel *et al.*, 2012) to support users’ ability to disseminate models in a diverse array of modeling formalisms. In addition, as SBML is updated and novel modeling techniques arise, the active, open-source community behind JSBML will continue to provide a comprehensive, computable interface for systems biology models.

## Supplementary Material

Supplementary Data
