# Supplementary material for: JSBML 1.0: providing a smorgasbord of options to encode systems biology models
Source: Bioinformatics. 2015 Jun 16;31(20):3383–6. doi: 10.1093/bioinformatics/btv341 (PMC4595895; doi:10.1093/bioinformatics/btv341)
Supplement: Supplementary Data [file supp_31_20_3383__index.html]

JSBML 1.0: providing a smorgasbord of options to encode systems biology models — JSBML 1.0: providing a smorgasbord of options to encode systems biology models — JSBML 1.0: providing a smorgasbord of options to encode systems biology models — Supplementary Data 

# JSBML 1.0: providing a smorgasbord of options to encode systems biology models

## Supplementary Data

files

- Supplementary Data - pdf file
