# Supplementary figures and images for: JSBML 1.0: providing a smorgasbord of options to encode systems biology models
Source: Bioinformatics. 2015 Jun 16;31(20):3383–6. doi: 10.1093/bioinformatics/btv341 (PMC4595895; doi:10.1093/bioinformatics/btv341)

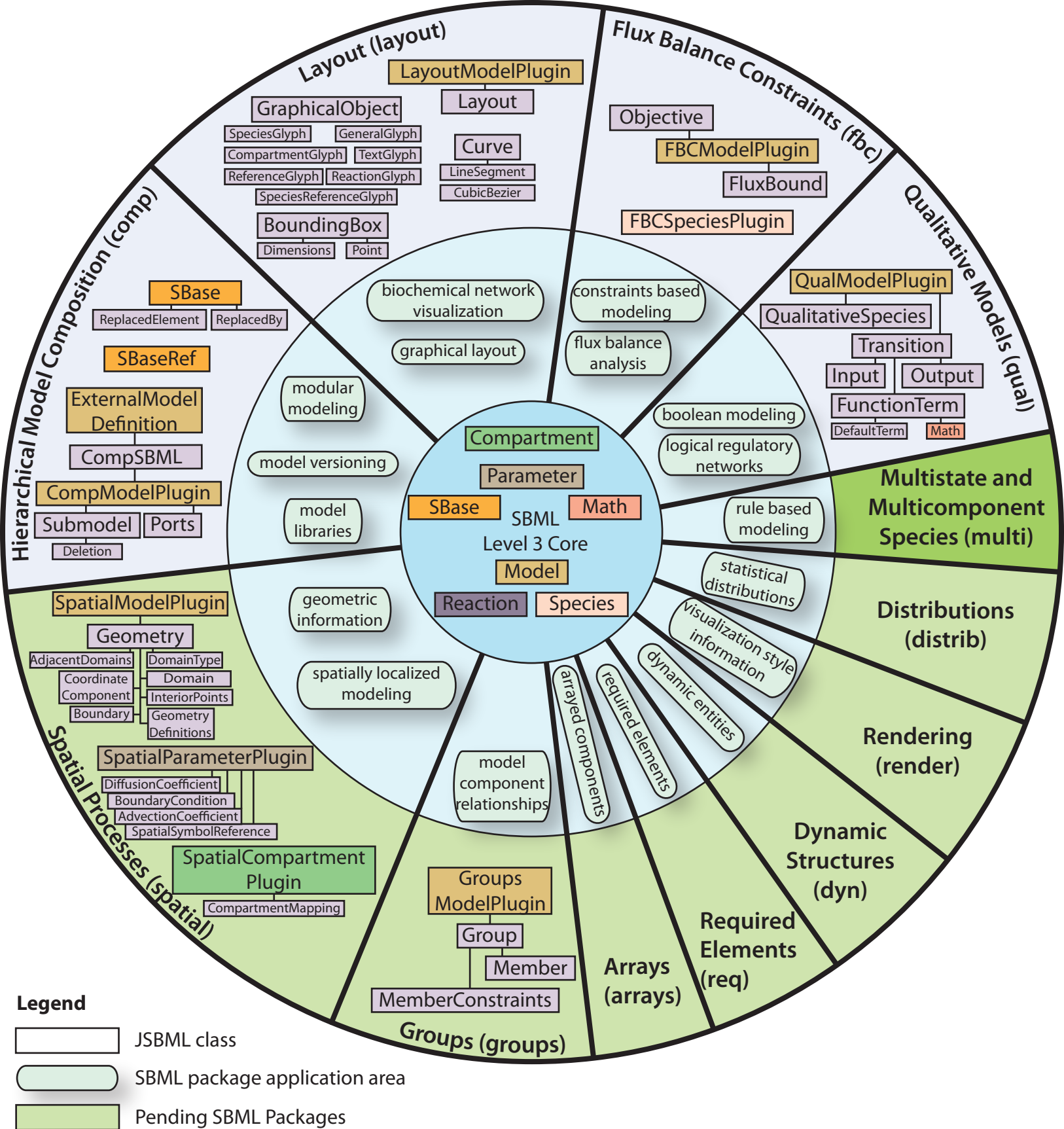

Supplement: Supplementary Data [file supp_btv341_sbml_lvl3_extensions.pdf]
